# An introduction of preference based stepping ahead firefly algorithm for the uncapacitated examination timetabling

**DOI:** 10.7717/peerj-cs.1068

**Published:** 2022-09-02

**Authors:** Ravneil Nand, Bibhya Sharma, Kaylash Chaudhary

**Affiliations:** The University of the South Pacific, Suva, Fiji

**Keywords:** Optimization, Swarm intelligence, Meta-heuristic algorithm, Stepping-ahead, Preference, Uncapicitated exam timetabling problem

## Abstract

In recent times, there has been a growing attention to intelligent optimization algorithms centred on swarm principles such as the firefly algorithm (FA). It was proposed for the continuous domain that mimics the attraction of fireflies to flashing light and has been used in discrete domains *via* modification. A discrete domain that is a major challenge in most higher education institutes (HEI) is examination timetabling. This article presents a new methodology based on FA for uncapacitated examination timetabling problems (UETP) where the proposed method is an extension of earlier work by the authors on the continuous domain. UETP is considered in this article as it is a university examination timetabling problem, which is still an active research area and has not been solved by FA algorithm as per authors knowledge. The proposed method concentrates on solving the initial solution using discrete FA where it consolidates the reordering of examinations and slots through a heuristic ordering known as neighborhood search. Three neighborhoods are employed in this research, where one is used during the initialization phase while two are utilized during solution improvement phase. Later, through preference parameters, a novel stepping ahead mechanism is used, which employs neighborhood searches built on previous searches. The proposed method is tested with 12 UETP problems where the preference based stepping ahead FA creates comparative results to the best ones available in the literature for the Toronto exam timetabling dataset. The results obtained are proof of concept at the preliminary stage and require further experiments on other educational datasets such as the second international timetable competition benchmark sets. The newly introduced preference based stepping ahead mechanism takes advantage of the current best solution space where it exploits the solution space for better solutions. This paves the way for researchers to utilize the mechanism in other domains such as robotics, *etc*.

## Introduction

Computer Science has seen a vast range and magnitude of research in the areas that leverage on information communication technology (ICT) such as designs and architecture (Sharma et al., 2019; Eden & Hirshfeld, 2001; Gharehchopogh, Farnad & Alizadeh, 2021a), models, facilitation, forecasts and predictions (Nand, 2016), teaching and learning, tools and technologies (Sharma, Vanualailai & Singh, 2017), and diagnostics (Baraldi, Bonfanti & Zio, 2018; Shishavan & Gharehchopogh, 2022). ICT is now more and more focused on optimizing the resources to efficiency. The use of optimization techniques (Gharehchopogh, 2022b; Gharehchopogh & Abdollahzadeh, 2021; Mohammadzadeh & Gharehchopogh, 2021) provide improvements in the areas where function objectives are to be solved to optimality or near optimality (Chen et al., 2015). Finding the global minimum or maximum depending on the objective(s) and restrictions, if any, is called optimization (Deb et al., 2002; Gharehchopogh, 2022b). Any problem having multiple objectives (Abdollahzadeh & Gharehchopogh, 2021) there comes a new level of complexity, since all or almost all of the objectives need to be met, and the optimal region would alter dramatically. Therefore, smart or intelligent algorithms are needed to tackle complex problems where multiple objectives and/or constraints are present.

In addition to, while dealing with complex real-world problems like designing solutions and/or time-series prediction, it is very important to optimize solutions either using selected optimizer (Ghafori & Gharehchopogh, 2021; Gharehchopogh, 2022a) or apply feature selection to remove noise (Abdollahzadeh & Gharehchopogh, 2021; Mohmmadzadeh & Gharehchopogh, 2021; Naseri & Gharehchopogh, 2022; Gharehchopogh, Maleki & Dizaji, 2021b). The education sector also requires optimal solutions for assorted task which range from website response rate (Yunfeng, 2010) to student performance (Nand, Chand & Naseem, 2020a). In higher education institutes (HEI), one of the fundamental areas that need optimization is scheduling. Scheduling is the process of efficiently arranging, regulating, or optimizing items (Baker & Trietsch, 2013). An area of scheduling present in HEI is timetabling. It is not only in HEI but secondary education, transportation companies, sport institutions, health, and aviation, have to deal with timetabling issues on a regular basis throughout the year. Timetabling is a NP-hard problem (Bilgin, Özcan & Korkmaz, 2006; Burke et al., 2004), which requires assigning limited resources to a task over period of time in relation to an organization’s operating rules and needs. This is a method that assigns courses or subjects to specified time slots or periods (Daskalaki & Birbas, 2005; Daskalaki, Birbas & Housos, 2004). According to Babaei, Karimpour & Hadidi (2015), the categories of methods for solving the timetabling problems are operational research techniques, multi-objective/criteria methods, intelligent novel approaches, metaheuristic algorithms, and distributed multi-agent systems.

Exam and course timetabling are the two types of university timetabling problem (Bilgin, Özcan & Korkmaz, 2006; Burke et al., 2004; Eley, 2006). In the current research, the Examination Timetabling Problem (ETP), a highly constraint problem (Burke et al., 2004; Bilgin, Özcan & Korkmaz, 2006) is considered. ETP (Burke et al., 2004) is dynamic in nature that requires spreading exams within a set of available time slots (Eley, 2006). The goal is to space exams equitably across the time frame, taking into account student workload and any constraints. The hard and soft constraints are the two types of constraints to solve. Hard constraints, such as room capacity, cannot be violated, whereas soft constraints are desired but can be violated such as a student should not seat for two exams on the same day. While different researchers have used different techniques including conventional algorithms and computational intelligence approaches to solve exam timetabling, the graph coloring approach was one of the very first. Carter, Laporte & Lee (1996) employed a variety of heuristic orderings formulated on graph coloring to sort the examinations. As an improvement, Burke et al. (2010) has demonstrated the efficacy of the meta-heuristic technique in which variable neighbourhood search was utilized to address the problem. Generally, ETP involves assigning examinations to a limited number of time slots and rooms while adhering to a set of hard constraints and attempting to minimize soft constraint violations, whereas time is not an essential constraint in real-world scheduling (Abdullah et al., 2007).

In the past few years, there has been a growing interest in timetabling problems (Zhu, Li & Li, 2021; Aldeeb et al., 2019). The examination timetabling problems are classified into two categories: Capacitated Examination Timetabling Problems (CETP) and Uncapacitated Examination Timetabling Problems (UETP). UETP ignores the need for room capacity and the only hard constraint is that a student cannot take two exams at the same time. While, the only soft constraint is to spread examinations out as evenly as possible, where penalty is applied on the distribution. In the literature, exams ordering include random order (RO), largest degree (LD), largest weighted degree (LWD), largest penalty (LP), and saturation degree (SD) (Burke, Qu & Soghier, 2014; Rahman et al., 2014; Mandal & Kahar, 2020). These are referred to as graph heuristics ordering strategies (Carter, Laporte & Lee, 1996).

One metaheuristic algorithm that has still not been applied to UETP as per the authors knowledge is the firefly algorithm (FA). This is mainly due to the algorithm’s application nature, which has been restricted to the continuous domain (Abedi & Gharehchopogh, 2020; Zaman & Gharehchopogh, 2021; Goldanloo & Gharehchopogh, 2022). In 2009, Yang (2009) proposed the FA scheme, a swarm intelligence algorithm based on grouping behaviour of fireflies. For its stochastic search characteristic, the algorithm is frequently employed in a variety of disciplines. FA is commonly employed in local search since it allows for better exploitation (Aydilek, 2018). FA has the advantage of not having a single best or a single global best, which keeps it from becoming stuck in early convergence or local minima (Fister et al., 2013). There have been many modifications of FA to suit the needs of the problem, one such modification is its application from continuous domain to discrete (Tilahun & Ngnotchouye, 2017).

In recent years, new modifications to FA such as elitist and binary firefly algorithms, chaos-based firefly algorithms, and parallelized firefly algorithms has been proposed to improve the performance (Surafel & Hong, 2012; Wang et al., 2012; Fister et al., 2013). Each of these changes has improved FA’s capabilities, the algorithms’ reliance on the initial adjusting parameters remains, and these changes require extensive testing with a wide range of problems (Tilahun, Ngnotchouye & Hamadneh, 2019), especially the discrete domain. Therefore, to use the FA with ETP, the algorithm needs to be changed to discrete domain as ETP is a discrete domain problem. Some work on discrete FA has been used on different problems (Chhikara & Singh, 2015; Tilahun & Ngnotchouye, 2017) where the proposed discrete algorithm is used with discrete variables for solving optimization problems. The algorithm can change the solution in different domains, these modifications can typically be classified into two types: continuous change and discrete change (Tilahun & Ngnotchouye, 2017). In continuous change, the modification is done in the continuous space and a discrete mechanism is employed to convert the solution to discrete, while in discrete change, the modification is performed directly on discrete domain. Switching domains is not required in the discrete change approach. Some of the modifications in continuous space, as discussed in detail in Tilahun & Ngnotchouye (2017), uses Sigmoid function, Tan hyperbolic function, rounding, random key, and problem based approach. As for modifications done directly in discrete space include problem based approach, entry replacing, crossover, sequential, copying, and systematic approach. UETP requires standard FA to either do modifications in continuous domain or discrete domain where the solution is in discrete as to suit the problem.

The primary goal of this research is the implementation of the discrete modified FA to solve the Uncapacitated Examination Timetabling Problem (UETP) as there is scope to firstly improve discretization of FA and UETP is still very active research area. The discrete modified FA is coined the term Preference-based Stepping ahead firefly algorithm. Discrete Stepping ahead mechanism is an extension of earlier work on the continuous domain by the authors (Nand, Sharma & Chaudhary, 2021; Nand, Chaudhary & Sharma, 2020b). The implementation first improves the proposed algorithm by providing better exploration through the neighbourhood searches and subsequently uses Stepping Ahead mechanism based on preference. Preference incorporates the ability to decide when to initiate the stepping-ahead mechanism. FA was also selected based on its superior performance than other common meta-heuristics algorithms (Hassan & Rashid, 2020b, 2020a; Nand & Sharma, 2019).

In this research, the preference based stepping ahead FA is discussed with application to exam timetabling problems. The following are the three primary contributions of this study:

- Discretization of FA: The algorithm is modified from continuous domain (Yang, 2009) to discrete domain through use of partial exams and heuristic orderings to solve exam timetabling problem. The model generates feasible exam timetabling solution for uncapacitated examination timetabling problem. The discretization of FA gives opportunity to researchers to make use the phases of the discretization on any algorithm to be used for exam timetabling.
- Preference-based Stepping ahead Model: A novel method for searching a larger search space in terms of exploration in order to locate better candidate solutions. Here, the algorithm is modified from authors previous work on continuous domain (Nand, Chaudhary & Sharma, 2020b; Nand, Sharma & Chaudhary, 2021) to discrete, to be used with examination timetabling problem. There was a limitation on when to initiate or activate the stepping ahead criteria, therefore, preference mechanism is utilized. The fireflies are searching in terms of steps where new neighbourhoods are used together with previous solutions through preference operated. This will pave the way for the researchers to explore preference and stepping ahead mechanism in different domains such as health, sports and transportation.
- Performance analysis: The proposed method is tested on 12 test problems of Uncapacitated Examination Timetabling Problem and later the results are statistically compared with the similar methods from the literature. The results are comparative and stepping ahead allows to narrow the gap in the literature where methods get stuck on local minima and hardly get chance to move out from it.

The following is how the rest of the article is organized: The literature is discussed in “Related work”, and the problem formation is outlined in “Problem formulation: uncapacitated examination timetabling”. “Proposed algorithm: preference based stepping ahead firefly algorithm” displays the proposed algorithm and the experimental setup is discussed in “Experiments”. “Results and discussion” presents the result findings with discussion, while “Conclusion and future works” concludes the article with a discussion on future research.

## Related work

This section gives an overview of the algorithms that are related to the proposed technique. Firstly, selected modifications of FA to the discrete domain are presented and later uncapacitated examination timetabling problem is discussed together with limitation and contribution.

### Modification of FA to discrete domain

Firefly algorithm (FA) is a meta-heuristic algorithm based on swarm intelligence introduced by Yang (2009) for continuous optimization problems. Since then, FA has seen numerous modifications and hybridizations for the algorithm inspired by the flashing characteristics of fireflies during night, showing promising results in vast domains and applications (Wang et al., 2015; Chou & Ngo, 2017) with discrete domains (Tilahun & Ngnotchouye, 2017) being no exception.

In 2011, Gandomi, Yang & Alavi (2011) proposed a modified FA to solve classical structural optimization problems where integer valued variables were utilised. Rajalakshmi, Subramanian & Thamizhavel (2015) modified FA algorithm using the sigmoid function for application to optimal radial distribution reconfiguration using distributed generators. Similarly, Chhikara & Singh (2015) presented a discrete FA for improvement in Blind Image Steganalysis. The improved version was proposed in Chhikara, Sharma & Singh (2018). In addition, Crawford et al. (2015) proposed a Modified Binary Firefly Algorithms (MBFF) to solve Set Covering Problem (SCP) using different transfer functions. Recently, a modified FA was utilized for solving a Vehicle Routing Problem (VRP) variant (Trachanatzi et al., 2020). The modification saw encoding/decoding process through Cartesian coordinates of each node.

The encoding/decoding process has not been updated only on the continuous domain but this can also be seen to take place in the discrete space (Baykasoğlu & Ozsoydan, 2015). In (Baykasoğlu & Ozsoydan, 2015), the authors used a modified FA in a multiple step process to solve the mechanical design optimization problems. Priority based encoding was utilized with random flight and local search. Zhu, Zhang & Wang (2018) have used discrete FA termed as Pareto firefly algorithm to solve disassembly line balancing problem where swap operation was utilized. More recently, authors of Carbas (2020) proposed enhanced FA to solve discrete nonlinear programming problems. A review article focusing on discrete FA appeared in Tilahun & Ngnotchouye (2017), detailing continuous and discrete space transformations.

The modifications have proved to be good solutions to the applied problems, however, the proposed approach in the current research differs from the existing work in the following ways for uncapacitated examination timetabling problem:

1. A stepping ahead mechanism is utilized where best candidate solution is used to search for solutions and later the found solution if not better or closer to the threshold is used to search for new solutions until the required generation.
2. A preference mechanism is used to activate the stepping ahead, where stepping ahead mechanism is only used if the solution is not improving for few generations.

### Uncapacitated examination timetabling problem

One type of ETP that is still actively researched is the uncapacitated examination timetabling problem (UETP). Carter, Laporte & Lee (1996) proposed the problem in 1996, that stated 13 challenging real-world problems, which were mostly from universities in North American, commonly known as “Toronto instances”. The authors proposed the graph heuristics ordering strategies to solve the 13 problems, however, it was time expensive.

In the literature, there are several ways to solve the UETP, the best and recent ones are discussed in this section. Yang & Petrovic (2004) proposed a fuzzy set similarity measure to show effectiveness of Case Based Reasoning (CBR) methodology. In (Burke & Bykov, 2008), Burke and Bykov proposed a new local search strategy named late acceptance and which was based on hill climbing technique. The strategy made the simple hill climbing algorithm to perform better with late acceptance. In 2010, Burke et al. (2010) introduced variable neighbourhood search where it was hybridized with genetic algorithm to solve UETP and the performance was relatively better in most of the benchmark problems. Fong, Asmuni & McCollum (2015) introduced a variant of artificial bee colony (ABC) algorithm with Great Deluge (GD) to solve the UETP. The idea was to enhance the ABC algorithm’s ability to explore and exploit the search areas.

More recently, Burke & Bykov (2016) proposed a flexible GD metaheuristic approach based on flexible acceptance condition to solve UETP. The method outperformed the authors earlier work on GD approach. In order to confine the exploration to the feasible solution space, Leite et al. (2018) developed a population-based algorithm that uses the threshold acceptance local search metaheuristic. Mandal & Kahar (2020) carried out a hybridization of two techniques, graph heuristics and major trajectory metaheuristics. This resulted in multiple algorithms exploited to gave best performance in capacitated and un-capacitated examination timetabling problems. Similarly, Bellio et al. (2021) used a two phase multi-neighborhood simulated annealing approach to solve UETP. The results are competitive with known best results of UETP.

### Limitation and contribution

This article is introducing a preference-based stepping ahead firefly algorithm for solving examination timetabling problem. The main issue found through the comparative study was that meta-heuristic algorithms needed some form of hybridization to solve the UETP. Some techniques are either hyper heuristic methods or uses hybridization to solve the problems. The proposed method in this research is not hybridization, however, two relatively new mechanisms are used to demonstrate how solution can be improved without using hyper heuristic methods. The technique used to solve UETP can be applied to other domains as well whether it is continuous or discrete domain.

## Problem formulation: uncapacitated examination timetabling

In the following section, the Toronto Uncapacitated Examination Timetabling Problem (UETP) is described.

The UETP formulation was adapted from Burke et al. (2004) and the terms are defined as follows:

- *N* denotes the number of exams
- *P* denotes the number of periods
- *M* denotes the number of students
-
$E = \{ {e_1},...,{e_n}\}$ denotes the set of exams
-
${({C_{ij}})_{E \times E}}$ is the conflict matrix, where each element in the matrix represents the number of students taking the exam *i* and *j*, and where $i, j \in {{\{}1, ..., E{\}}}$
-
${t_k}(1 \ge {t_k} \ge T)$ is the time slot associated with exam $k\ (k \in E)$

One soft constraint in the UETP is that a student cannot take two tests in nearby periods that are one to five slots apart. The function ${f_c}$ represents the soft constraint. The problem formulation is provided by Eq. (1):

(1) $$minimise\quad {f_c} = \displaystyle{1 \over M} \cdot \sum\limits_{i = 1}^{|N|{\rm } - 1} {\sum\limits_{j = i + 1}^{|N|} {{C_{ij}}} } \cdot proximity(i,j)$$

(2) $$\eqalign{& where \cr & proximity(i,j) = \left\{ {\matrix{ {{2^{5 - |{t_i} - {t_j}|}},} & {{\rm if1} \le {\rm |}{{\rm t}_{\rm i}}{\rm - }{{\rm t}_{\rm j}}{\rm |} \le {\rm 5}.} \cr {0,} & {{\rm otherwise}.} \cr } } \right.}$$

(3) $$\eqalign{& {\rm subject\ to} \cr & \sum\limits_{i = 1}^{|N{\rm |} - 1} {\sum\limits_{j = i + 1}^{{\rm |}N{\rm |}} {{C_{ij}}} } \cdot \lambda ({t_i},{t_j}) = 0,\quad \lambda ({t_i},{t_j}) = \left\{ {\matrix{ {1,} & {{{t}_{i}}{\rm\ =\ }{{t}_{j}}.} \cr {0,} & {{{t}_{i}} \ne {{t}_{j}}.} \cr } } \right.}$$

The penalty for scheduling exams ${e_i}$ and ${e_j}$ in the periods ${t_i}$ and ${t_j}$, respectively, is given by Eq. (2). For exams that are one to five periods apart, the penalty weighting factor is 16, 8, 4, 2, and 1, respectively. For exams that are more than five periods apart, there is no penalty weighing factor. The hard constraint (Eq. (3)) states that there must be no conflicts between exams scheduled during the same period. This cannot be violated as the solution will not be a feasible one.

## Proposed algorithm: preference based stepping ahead firefly algorithm

This sections starts with operators used for discretization of the Firefly algorithm and then moves to implementation.

### Feasible solution

For any algorithm to solve a given problem, it first needs to align its solutions in terms of the given problem. The same is for the exam timetabling problem. To apply FA to optimize the solution, it first needs to have the feasible solution. This is where the discretization of the solution occurs for the FA. To find the feasible solution, a lot of techniques are available in the literature, but the one used in this research is partial exam technique with exam swap move. Partial exam technique allows to solve the problem faster, and it has been used previously to solve UETP successfully (Mandal, Kahar & Kendall, 2020). During the initialization process, partial exam technique is used to find the feasible solution. Exam timetabling problems have seen the use of graph heuristics ordering strategies where the constraints are sorted according to different techniques. The techniques include the largest degree (LD), largest weighted degree (LWD), largest penalty (LP), saturation degree (SD) and random order (RO) (Carter, Laporte & Lee, 1996). In this study, LD is used where the exams are arranged in descending order of the number of conflicts between each exam and the others. Exams that produce infeasibility are given priority since they are logically difficult to assign into the schedule and hence are handled first. The partial exam approach utilizes partial graph heuristic orderings where 5–10 percentage of the sorted exams are taken and assigned to the slots where there is no conflict. The selection of slots are kept at random to give a better spread. It then increments the process by earlier percentage until no exam is left to assign. If a feasible solution is not obtained, the optimization operator such as the exam swap operator is applied to make it feasible; move number 0 as shown in Table 1. The exam swap operator moves the non-allocated exams to random slots and any conflicting exam is removed and later those exams are placed in another random slot and it continues until no exam is left.

**Table 1:** Search space exploration.

Number	Moves
0	Swap move
1	Traditional Kempe Chain
2	Random Kempe Chain with swap move

### Supported neighborhoods

Feasible solution generated is able to solve the hard constraint, which stipulates that there must be no conflicts between exams scheduled during the same time period. The mechanism known as neighborhood search is utilized for the discretization of FA, where it is intended to improve the quality of the solution *via* stochastic process. Now, the solution needs to be optimized or improved in terms of the soft constraints. The solution space is now discrete after using graph heuristic orderings, therefore, to solve soft constraints neighborhood operators are needed. The proposed method supports two neighborhoods that the firefly algorithm selects in each iteration to generate a candidate of solutions. These neighborhoods are called “moves”. The moves are based on Kempe Chain. Both moves perform small transformations to the incumbent solution with relation to current cost. The moves are presented as moves 1 and 2 in Table 1.

Move 1 shown in Table 1 is the Traditional Kempe Chain, originally employed to solve the “four color problem”. A number of chains consisting of exams from either of the two periods are built using the two sets of exams allocated to the two periods. It begins by shifting one element at a time from one random timeslot to another. To maintain feasibility, any conflicts through these moves are solved by moving back and forth exams until no conflicts are present in the two time slots. Move 2 is the Random Kempe Chain with swap move. It is same as the traditional Kempe chain movement but with difference that only 1 random exam is exchanged and to maintain feasibility, if the moves are not able to find feasibility in 20 exchanges, a swap move occurs where both timeslots are exchanged.

In circumstances where a search technique exhibits diverse behaviors in response to different movements, the above concept is valid. Moving high conflicting exams more frequently results in infeasibility and/or a bigger average increase in the cost function than moving low conflicting exams in most real-world timetabling problems. As a result, some moves are more commonly accepted than others (with the same acceptance condition for all moves). In this situation, the search can be viewed of as biased toward finding a better solution for a small number of variables (exams), but it may overlook opportunities to explore large portions of the search space, resulting in never finding even better solutions. Therefore, an operator called preference is utilized. Here, a second move is only used with the stepping ahead operator, and when the algorithm assumes it is stuck in a local optima it goes back a few iterations and restarts neighbourhood search with previous best solutions. The number of iterations can be based on user preference and/or problem dependent, therefore, 10 iterations back from where an improved solution was seen is utilized.

### Modification of FA

Algorithm 1 is a modified version of the original firefly algorithm that has been used in this study. Algorithm 1 is modified to discrete domain with preference mechanism from the one given in the previous work of the authors (Nand, Chaudhary & Sharma, 2020b; Nand, Sharma & Chaudhary, 2021). The modification of the algorithm is from continuous domain to discrete to suit uncapacitated examination timetabling problem. The computation complexity of the algorithm is $O({n^2})$, where n is the number of fireflies. The fireflies and state variables are initialized using the greatest degree (LD) constraint sorting method in Step 1 of the algorithm. For the population array, a random solution is chosen. In Step 2, the new candidate solutions are evaluated. The objective function evaluates the total population in terms of the fitness of all the fireflies. Here, the neighbourhood operators are utilized which are based on the Kempe chain technique. Initially, any solution is selected that is not the same as the current selected solution and this is continued till five cycles. The Kempe Chain move is selected first, while step 3 is only triggered when no progress is achieved using the move for 10 generations.

**Algorithm 1:** Proposed discrete stepping ahead firefly algorithm.

**Step 1:** Initialize population of fireflies
Random population of N solutions using LD constraint sorting
Each exam is placed in non-conflicting slot
Initialize all variables
**while** *t* < *MaxGeneration* **do**
** for** *i* = *1: n (all n fireflies)* **do**
** for** *j* = *1: n (all n fireflies)* **do**
** Step 2:** Evaluation
** if** * ${I_j \leq I_i}$* **then**
move firefly in relation to i using the Kempe move; $I_{new}$
** while** *count* $\leq$ *5* & *Ii* = *I_new_* **do**
move firefly in relation to i using the Kempe move; * $I_{new}$*
** end**
** end**
** Step 3: Stepping ahead**
** if** *Stepping ahead is activated* **then**
move firefly in relation to j using the Kempe move; $I_{new}$
** while** *count* $\leq$ *5 &* $I_{new} - I _j\gt Q\ ||\ I_i= I_{new}$ **do**
move firefly in relation to $I_{new}$ using the Kempe move 1 & 2; $I_{new}$
** end**
** end**
** end**
nothing
** end**
Rank fireflies and update best using threshold and probability; Preference utilized;
** if** *No improvement* **then**
Stepping ahead is activated;
** else**
Stepping ahead is deactivated;
** end**
** if** *No improvement due to stepping ahead* **then**
restart 10 steps back where there was improvement;
** end**
**end**
**Post processing the results and visualization;**

Step 3 was introduced so that the algorithm can avoid getting stuck in the local optima. In step 3, if the solution of firefly *i* with respect to firefly *j* does not improve in terms of the fitness function over 10 generations, the algorithm looks for a new solution further in the space area of firefly *j*. The term coined to for the algorithm is *Preference based Stepping Ahead* (Nand, Chaudhary & Sharma, 2020b; Nand, Sharma & Chaudhary, 2021). The mechanism uses new solution as input of next move to find newer solution. The idea is not to use best solution rather any solution apart from the current best so that new solution that is better can be found in steps where there may be worst solutions before the global best.

**Figure 1:**
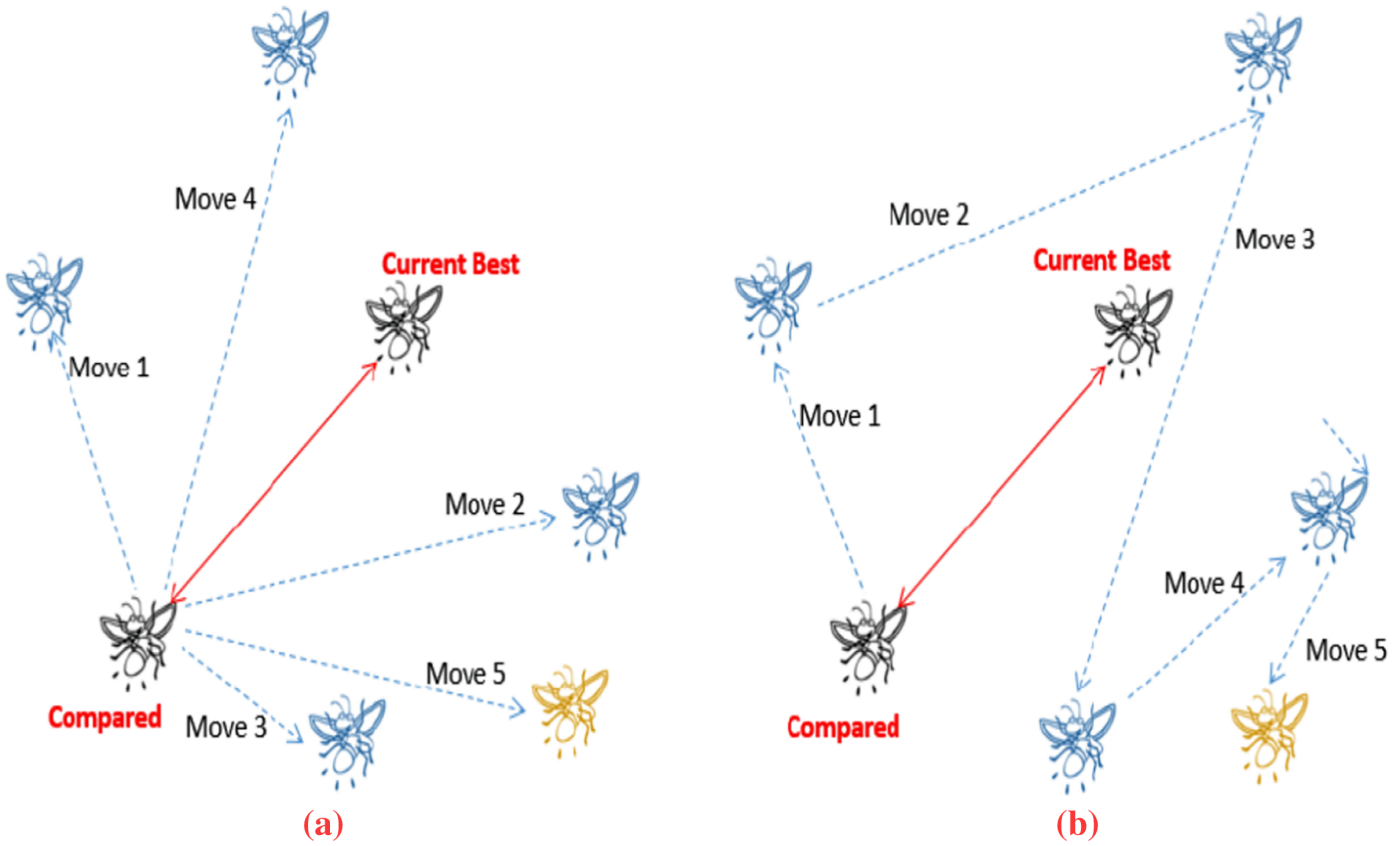
Firefly movement (adopted from Nand, Sharma & Chaudhary (2021)). (A) Shows typical moves for FA. (B) Shows stepping ahead move for FA.

Figure 1 depicts how a firefly ( $X_i^t$) goes from the present location to the new best position ( $X_i^{t + 1}$) in terms of the current best firefly position ( $X_j^t$), demonstrating the stepping ahead feature. The figure shows the typical movement where at least five moves are utilized to find the best solution. Figure 1A shows how a standard firefly would look for the better solution while Fig. 1B shows the movement based on the stepping ahead mechanism. Each step taken is based on previous step to allow worst solutions to guide the search space.

The goal here is to show how a point in a search space with a lower fitness value might lead to the global optimal solution. If the FA population is not adequately controlled, a greedy method will likely to eliminate promising spots in the early generations. When fireflies will get closer, the algorithm will still hunt for solutions that are further away from the best discovered, which effectively gives the algorithm a chance to get out of local minima as local minima’s can sometimes make algorithms belief it has found the global minima for instance for complex problems.

Finally, the ranking of solutions is carried out and the steps are repeated until maximum execution time has reached. It is natural for an algorithm to seek for the best solution, but for exploration, a better approach that can provide optimal solutions when the problems get complicated is required. It is known, normally the best solution could be surrounded by worst ones, therefore, while accepting solutions, two steps are taken when new solution is not better than previous. Firstly, a threshold difference is used which is called *Q* and it is reset every time stepping ahead is activated. From ${Q_{max}}$ to ${Q_{min}}$. ${Q_{min}}$ is a smaller difference to allow accepting to only closer points in the neighbourhood. Secondly, probability is used as well where the solutions are only accepted if the yield is less than the rand generated number. Here the bad solutions are accepted based on Eq. (4):

(4) $$rand \le {e^{ - (({x_{new}} - {x_i}/{x_i})/T)}},$$

where *rand* returns a single uniformly distributed random number in the interval (0,1). In Eq. (4), ${x_{new}}$ is the new position of firefly *i*, ${x_i}$ is the current position of the best firefly *i* and *T* is the current light intensity. Also, *T* changes in every generation as it is the product of mutation coefficient and current *T* for the generation.

### Detailed execution procedure

The overall framework of the FA-Step approach for the Uncapacitated Examination Timetabling Problem is based on three steps:

- Step 1 – *Find Feasible Solutions*: The solution is constructed using partial exam assignment strategy together with the graph heuristic orderings. Largest Degree (LD) graph heuristic orderings is used to sort the exam based on the constraint. In LD, exams are sorted in descending order according to the number of conflicts each exam has with the others then using the partial exam technique the sorted exams are placed in non-conflicting slots. This ensures finding the feasible solutions during initilization by placing the exams in different slot based on degree on conflict of exams.
- Step 2 – *Apply Kempe Chain Move*: After the feasible solution is found, it needs to be improved. This improvement comes from using the neighborhood operators. The Kempe Chain move is used to improve the current best solution.
- Step 3 – *Apply stepping ahead mechanism*: The main feature of the algorithm is to use step ahead technique with preference. Here Kempe Chain moves are used to improve the solution where if the first move does not find different solution than current best it advances to use second move and repeats. However, the next move uses the solution provided by previous move. This is orchestrated by utilizing a promising solution found in a previous move, with each move attempting to improve it and forwarding the result on to the next. The activation of the mechanism is done through preference operator.

**Figure 2:**
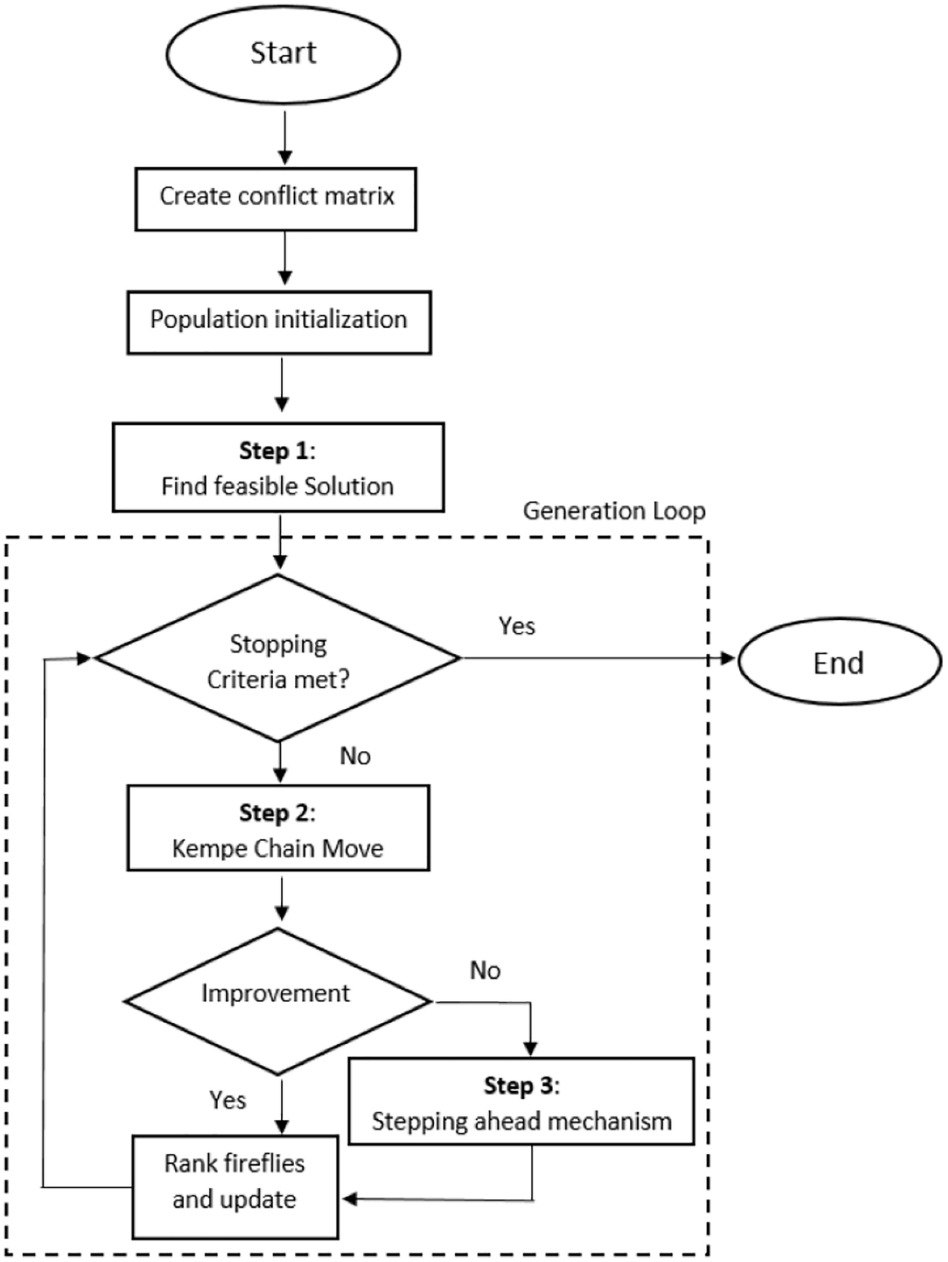
Flowchart of the proposed method.

In order to solve the UETP, the proposed algorithm in this research is split into three processes as stated above. Through the initialization phase, the goal is to provide an initial solution which is later improved by applying phases 2 and 3. Phase 2 uses simple moves to improve the solution and later after few iterations phase 3 is enforced, where neighbourhood operators are used as steps for improvements in iterative manner. The flowchart of the proposed algorithm is shown in Fig. 2.

## Experiments

The experimental setup and results analysis for the proposed algorithm are shown in this section. The experimental setting showcases the algorithm’s settings as well as the details of the Uncapacitated Examination Timetabling Problem (UETP). The mean, median, best, and worst findings of the experiments are presented in the Results section. The discrete FA will now be referred to as dFA, and the preference-based stepping-ahead discrete FA will be referred to as dFA-Step.

### Setting

The proposed algorithm was written in Matlab and tested on a laptop with the following specifications: Intel Core i7-8665U (CPU @ 1.90 GHz with 16 GB RAM) and Windows 10 operating system. The software will be made available to the researchers on request. The datasets are utilized same as in the literature without any modifications. This enables evaluation of the proposed algorithm’s true performance as described in the literature. Table 2 describes the parameters that must be set up for dFA. The parameter settings are based on published research. The dataset is described in depth in the upcoming subsection.

**Table 2:** Parameter setting.

Parameter	Value
Initial population size	50
No. of runs	10
Initial light intensity	0.1
Damping ratio	0.99
Light absorption coefficient	1
Attraction coefficient base value	2
Mutation coefficient	0.9
${Q_{min}}$	0.01
${Q_{max}}$	0.0001

### Dataset

Table 3 shows the characteristics of the 13 challenging real-world benchmark instances utilized in this research. The data were introduced by Carter, Laporte & Lee (1996) and can be retrieved from (Qu et al., 2009). The specific dataset has the property of very low learning curve with challenging nature, however, it has a relatively simple structure.

**Table 3:** Dataset problem characteristics (Toronto).

Problem	Exams	Students	Admissions	Density	Slots
CAR91	682	16,925	56,877	0.13	35
CAR92	543	18,419	55,522	0.14	32
EAR83	190	1,125	8,109	0.27	24
HEC92	81	2,823	10,632	0.42	18
KFU93	461	5,349	25,113	0.06	20
LSE91	381	2,726	10,918	0.06	18
PUR93	2,419	30,029	120,681	0.03	42
RYE92	486	11,483	45,051	0.07	23
STA83	139	611	5,751	0.14	13
TRE92	261	4,360	14,901	0.18	23
UTA92	622	21,266	58,979	0.13	35
UTE92	184	2,749	11,793	0.08	10
YOR83	181	941	6,034	0.29	21

## Results and discussion

This section presents the analysis of the proposed algorithm with discussion. Tables 4–6 reports the results obtained on benchmark dataset while Table 7 shows result comparison with works from the literature.

**Table 4:** Statistical summary of results on three dataset for dFA and dFA-Step. The bold results indicate the best results obtained on the datasets.

Algorithm	Instance	Best	Median	Worst	Mean
dFA	HEC92	10.31	10.58	**10.62**	10.51
	STA83	**157.03**	**157.05**	157.20	157.10
	YOR83	38.15	38.15	38.15	38.15
dFA-Step	HEC92	**10.17**	**10.40**	10.77	**10.42**
	STA83	**157.03**	**157.05**	**157.14**	**157.08**
	YOR83	**36.23**	**37.33**	**37.70**	**37.19**

**Table 5:** Results of Wilcoxon signed rank testing and ANOVA P-test for dFA-Step on three dataset.

Instance	dFA	*p*-value
HEC92	6.349e−01	6.63e−01
STA83	6.905e−01	8.24e−01
YOR83	2.381e−02	3.50e−02

**Table 6:** Statistical summary of results from dFA-Step.

Problem	Best	Median	Worst	Mean
CAR91	5.24	5.36	5.42	5.35
CAR92	4.41	4.49	4.58	4.48
EAR83	34.67	35.66	37.66	36.17
HEC92	10.17	10.40	10.77	10.42
KFU93	13.45	13.80	14.17	13.79
LSE91	11.28	11.30	11.80	11.47
PUR93	–	–	–	–
RYE92	8.71	8.85	9.40	8.94
STA83	157.03	157.05	157.14	157.08
TRE92	8.54	8.63	8.74	8.64
UTA92	3.62	3.69	3.74	3.69
UTE92	25.04	25.21	25.26	25.18
YOR83	36.23	37.33	37.70	37.19

**Table 7:** Best results from the literature compared with dFA-Step.

Algorithms	Car91	Car92	Ear83	Hec92	Kfu93	Lse91	Rye92	Sta83	Tre92	Uta92	Ute92	Yor83
dFA-Step	5.2	4.4	34.7	10.2	13.5	11.3	8.7	157.0	8.5	3.6	25.0	36.2
Alefragis et al. (2021)	4.6	3.8	32.7	10.0	12.9	10.0	8.1	157.0	7.9	3.2	24.8	35.1
Carter, Laporte & Lee (1996)	7.1	6.2	36.4	10.8	14	10.5	7.3	161.5	9.6	3.5	25.8	41.7
Merlot et al. (2002)	5.1	4.3	35.1	10.6	13.5	10.5	8.4	157.3	8.4	3.5	25.1	37.4
Yang & Petrovic (2004)	4.5	3.9	33.7	10.8	13.8	10.4	8.5	158.4	7.9	3.1	25.4	36.4
Abdullah et al. (2007)	5.2	4.4	34.9	10.3	13.5	10.2	8.7	159.2	8.4	3.6	26	36.2
Eley (2006)	5.2	4.3	36.8	11.1	14.5	11.3	9.8	157.3	8.6	3.5	26.4	39.4
Burke & Bykov (2008)	4.6	3.8	32.7	10.1	12.8	9.9	7.9	157.0	7.7	3.2	27.8	34.8
Burke et al. (2004)	4.9	4.1	33.2	10.3	13.2	10.4	–	156.9	8.3	3.3	24.9	36.3
Demeester et al. (2012)	4.5	3.8	32.5	10	12.9	10	8.1	157	7.7	3.1	24.8	34.6
Gaspero & Schaerf (2000)	6.2	5.2	45.7	12.4	18	15.5	–	160.8	10	4.2	29	41

In Table 4, the performance of dFA and dFA-Step methods are shown on selected three benchmark datasets. The selected datasets are smaller sized problems which can be solved within a short period of time. The bold results indicate the best results obtained on the datasets. It can be seen from the table that with stepping ahead mechanism, dFA-Step has improved performance in all the three datasets. Results are closer in STA83 dataset where mean and medium are same for both algorithms. dFA-Step has a consistent performance in all the datasets. Through the stepping ahead mechanism, results are fine tuned. For easy problems, dFA is self sufficient, but when it comes to complex datasets there needs to be some technique such as stepping ahead mechanism to guide the search space.

Wilcoxon signed rank testing (Derrac et al., 2011) was used in conjunction with a one-way ANOVA test to determine the significance level of dFA and dFA-Step performance. The test results are shown in Table 5 where the dFA-Step has a distribution similar to that of its counterpart for the first two instances, while it has significantly different distribution in YOR83. Additionally, the Anova *p*-value reported in Table 5 indicates there is no significance difference between the algorithms in HEC92 and STA83 while there is significance difference in YOR83 dataset. It is noted that the *p*-value is less than the threshold of 0.05 in YOR83 suggesting that the result distributions of the algorthims are significantly different.

The proposed method (dFA-Step) is significantly different than those of dFA in one out of three datasets experimented on. The proposed algorithm in this research article, dFA-Step, performed better due to the additional assistance through the newly proposed technique of preference based stepping ahead. The newly proposed algorithm improves the dFA by introducing the stepping ahead strategy, which improves the solution hunt, *i.e.*, exploration. In this case, the wider search space is considered, where the search space is the same but the search area is expanded by using the best known solution. The preference parameter controls whether the mechanism is activated or deactivated. This enables global search where local optima(s) can be avoided in some cases with a proper injection of good and bad solutions.

Table 6 displays the statistical results of dFA-Step on 12 University of Toronto benchmark exam timetabling tasks referred to as Toronto version I (Carter, Laporte & Lee, 1996). Version I was utilized for this research because it has been mostly reported in the literature and is a challenging dataset of real-world instances. The 13th dataset commonly referred to PUR93 is not discussed under results as it could not be successfully run due to its size, memory requirements and computational costs (Sharma, 2020) in the machine used for the experiments.

In addition, Table 6 shows a statistical summary of results from dFA-Step. Here it can be seen that the minimum and worst results are closer to each other indicating the consistent performance in multiple runs. The minimum, maximum, mean, and medium columns can be used to gather a number of valuable information about the algorithm. The medium column indicates the difference between the best and worst results while the mean value closer to minimum indicate a good distribution of the results.

In Table 7, the comparison of results of dFA-Step is carried out with algorithms from literature. These algorithms are widely employed to compare the performance of optimization algorithms on UETP. The results from the proposed method is in the row dFA-Step, while the other results are of heuristic methods from multiple metaheuristic variable neighborhood search framework (Alefragis et al., 2021) to a tabu search method implemented by Gaspero & Schaerf (2000). There are methods reported in the literature which utilize pre-testing phases or additional stages other than the stages used by dFA-Step, therefore, these methods are not reported in this research. As can be observed, the proposed algorithm produces similar results in 5 out of 12 cases. Further parameter tuning can also enhance the results.

The results of problems In Table 7, Hec92, Kfu93, Sta83, Ute92, and Yor83 are in the top five, while other results are in the upper half of the best results or in the top eight. It should be highlighted that dFA-Step has consistently delivered consistent results for all cases. It is very simple to experiment with the application of different algorithms and neighborhoods using the framework. In this research the focus was only on Kempe Chain; however, other neighborhood operators are available such as Move Worst Exam, Exam at Best Slot, Penalty Reducer, etc which can be utilized (Alefragis et al., 2021). The choice of moves have a considerable impact on the quality of the solutions generated.

**Figure 3:**
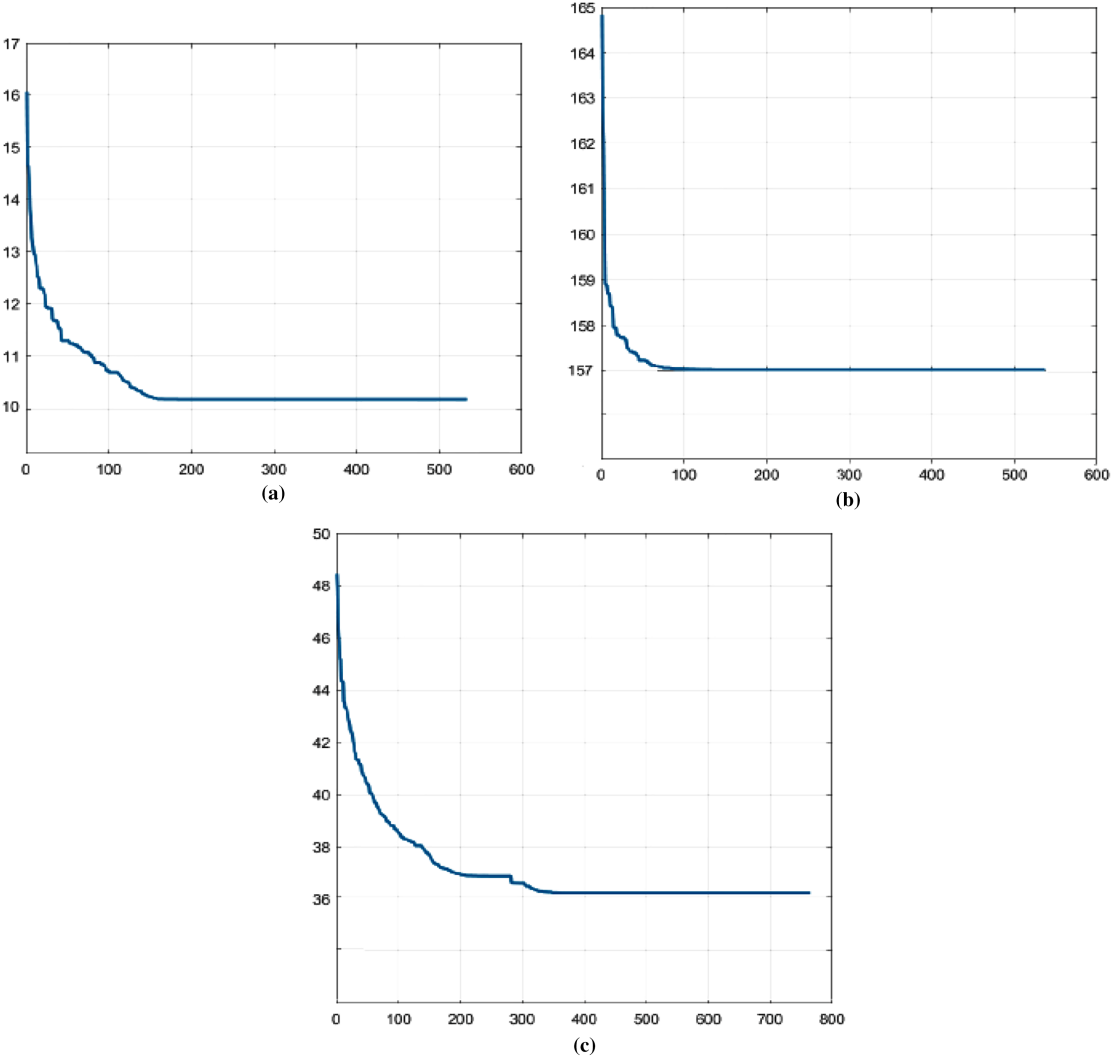
Average convergence graphs of dFA-Step. (A) HEC92. (B) STA83. (C) YOR83.

Figures 3A–3C display the average convergence graphs of dFA-Step. Figure 3A shows convergence graph of HEC92 dataset while Fig. 3B shows average convergence graph of STA83 dataset where uniform performance is shown after 100 or 150 generations. Similarly, Fig. 3C shows convergence of YOR83 dataset where it can be seen that the graph becomes uniform after around 180 iterations and later at 350 iterations.

Further analysis for dFA-Step can be made from the Figs. 3A–3C. It can be seen that the proposed algorithm was able to converge after 100 to 150 iterations. The figures show that when stepping ahead parameter is activated, there is swift change. The change is that, the mechanism drives the whole algorithm to better solution than its current state and a uniform distribution can be seen.

The results are preliminary proofs of concept, and more in-depth analysis is required to determine the best algorithm for solving such problems faster and with better results. The results are compared to the existing literature, which includes benchmark datasets, and, more importantly, the algorithms were tested on the same datasets without any changes. The two limitations of the research are as follows:

- Statistical comparison of different algorithms from the literature cannot be carried out which could have given a better insight of their structure.
- The source code of the algorithms from the literature is not readily available for testing.

Furthermore, the proposed method’s consistent performance was only attainable because of the addition of a preference-based stepping ahead parameter. The algorithm advances to a greater region of the search area for a better solution since it not only utilizes the best solutions to search ahead but also takes a proactive step where the worst solutions are used to guide the search space by preference mechanism activation. This gives opportunity to current researchers to explore the problems search space through stepping ahead. The main improvements of the proposed model in comparison to other methods in literature include the following:

- Using the stepping ahead mechanism allows a meta-heuristic algorithm to perform better as this is evident from results between dFA and dFA-Step.
- The preference operator can be used with hyper-heuristic models in literature to further enhance the performance.

One limitation of the proposed algorithm in other application is that the preference operator would need to be designed to suit the other application as currently it is designed for UETP.

### Convergence analysis

This subsection provides a theoretical exposition of the convergence of the proposed Preference based Stepping ahead Firefly Algorithm, based on the convergence criterion of stochastic algorithm (Solis & Wets, 1981) and proof provided on FA (Wei, Li & Zeng, 2021).

FA is seen to be a stochastic algorithm since it has stochastic process with state transition as highlighted by Wang & Song (2019). For any given minimization problem, where *f* is the objective function and *S* is the feasible solution, the result of optimization algorithm *O* in the *t*-th iteration would be ${x_t}$ (Wang & Song, 2019). Therefore, the next iteration will be ${x_{t + 1}} = O({x_t},\xi )$, where $\xi$ is the solution from the sample space. This holds true for any domain problem even discrete where *S* is a subset of ${R^n}$. The algorithm satisfies the following conditions:

1.
$f({x_{t + 1}})$ $\leq$ $f({x_t})$, and then $f(O({x_t},\xi ))$ $\leq$ $f(\xi )$,
2.
${\forall A \in S, s. t. v(A)\gt 0}$, $\prod\limits_{t = 0}^\infty {(1 - \mu (A))} = 0$,

where *v* is the Lebesgue measure, and $\mu$ is the probability measure.

**Lemma 1** (Solis & Wets, 1981) *When the algorithm O satisfies both conditions, then*, $\mathop {\lim }\limits_{t \to \infty } P({x_t} \in {R_{\varepsilon ,M}}) = 1$.

*That is, when t approaches* $\infty$, *the algorithm O converges to the global optimal solution domain* ${R_{\varepsilon ,M}}$ *with probability 1 where* $\varepsilon$ $\gt$ 0 and *M* $\lt$ 0.

**Lemma 2** (Wang & Song, 2019) *Since the number of iteration and individuals N are finite, the solution space S is finite as the boundary is less than* $\infty$.

*That means, each individual state and the firefly swarm state space is finite. The state of each firefly at iteration $t+1$ is only related to that of iteration t. Therefore, transition from state ${f(t)}$ to state $f(t + 1)$ only depends on iteration t state. So the firefly swarm state space $f(t)|t \geq 1$ is a finite Markov chain*.

**Theorem 1** *dFA-Step algorithm has global convergence*.

**Proof 1** *The fireflies update their positions as better new solutions are found. The proposed dFA-Step algorithm learns from the best solution:* $X_i^{t + 1}$ *= O(* $X_i^t$, $X_j^t$*), where i and j are firefly labels as shown in* Fig. 1. *The selection is only done when the new solution is better than previous solution. Therefore, the function value at iteration $t+1$ is smaller than that at iteration t. The condition 1 in the convergence criterion of the random algorithm is met*.

*Moreover, in dFA-Step, the state transition probability of iteration $t+1$ is only related to iteration t as seen in Lemma 2. The restart criteria in dFA-Step only restarts from previous state where the solution is better. Therefore, the probability of repeatedly missing the set S, when generating the solution is 0 as future states are based on current best state. The condition 2 in the convergence criterion of the random algorithm is met. That is, dFA-Step can converge to global optimal solution*.

## Conclusion and future works

The discrete firefly algorithm (dFA) was used in this study, and it was enhanced by incorporating a new and novel “preference-based stepping ahead” parameter to form a new technique dFA-Step to solve optimization problems known as uncapacitated examination timetabling problems (UETP). The dFA-Step was used to solve the 12 benchmark Toronto problems from the literature. The proposed technique outperformed the standalone algorithm (dFA) and a few selected methods from the literature, according to experimental results.

Through the use of the preference operator, the newly introduced preference-based stepping ahead method not only exploits the solution space but also explores beyond the best solutions or locations. This enables the algorithms to identify superior solutions that might otherwise be lost due to becoming trapped in the local optimum(s) and later switch to neighbourhood search to continue searching for more superior solutions. It is normal to say that at some given point in time, the best solutions are near to the worst solutions. Therefore, stepping ahead mechanism allows to take progressing solutions as inputs and generates new solutions. From the comparison to modified FA algorithm in the literature, it is evident that the proposed method works well on some of the selected datasets. This gives an opportunity to other researchers to explore further using the new preference based stepping ahead mechanism on other domains such as robotics and/or health.

The results are proof of concept at this early stage because its benchmark dataset based on timetabling problem at the University of Toronto (version I). Research in the future will concentrate on parameter adjustment to raise convergence and success rates even higher. Statistical analysis and benchmarking will also be conducted to support the method beyond the research environment as such comparison of the proposed method with some state-of-the-art techniques such as the fitness dependent optimizer and grey wolf optimization algorithm. Other benchmark datasets can be accommodated by the research such as having more constraints in the form of room allocations, etc.

## Additional Information and Declarations

### Data Availability

The following information was supplied regarding data availability: Data is available at The University of Nottingham: http://www.cs.nott.ac.uk/~pszrq/data.htm. Code is available at Github: https://github.com/ravneil/UnCapicitated-Exam-Timetabling.git.
